# What Are the Pearls and Pitfalls of the Dietary Management for Chronic Diarrhoea?

**DOI:** 10.3390/nu13051393

**Published:** 2021-04-21

**Authors:** Leigh O’Brien, Catherine L. Wall, Tim J. Wilkinson, Richard B. Gearry

**Affiliations:** Department of Medicine, University of Otago, Christchurch 8140, New Zealand; leigh.obrien@postgrad.otago.ac.nz (L.O.); catherine.wall@otago.ac.nz (C.L.W.); tim.wilkinson@otago.ac.nz (T.J.W.)

**Keywords:** chronic diarrhoea, diet, irritable bowel syndrome, FODMAP, SIBO, lactose intolerance, bile acid diarrhoea, sucrase-isomaltase deficiency, dietitian

## Abstract

Chronic diarrhoea affects up to 14% of adults, it impacts on quality of life and its cause can be variable. Patients with chronic diarrhoea are presented with a plethora of dietary recommendations, often sought from the internet or provided by those who are untrained or inexperienced. In this review, we summarise the possible causes of chronic diarrhoea that can be managed by diet, the symptom improvement and quality of life benefits but also the potential risks of such dietary treatments. Clinicians need to consider both the benefits and risks of dietary treatments before making dietary recommendations to manage chronic diarrhoea. The pivotal role that dietitians have in ensuring optimal symptom improvement without jeopardising nutritional and overall health is discussed.

## 1. Introduction

Chronic diarrhoea is variably defined but usually includes stools ranging from type 5 to type 7 on the Bristol stool form scale, a duration of more than four weeks, and frequency of stools (usually >25% of the time) [1]. The prevalence of chronic diarrhoea is difficult to determine due to these definition variations [2]. In a population of older adults, 14% were classified as having chronic diarrhoea based on increased stool frequency and the absence of abdominal pain [3]. However, this may include patients with structural or functional causes such as microscopic colitis or chronic diseases such as diabetes. A population-based study from the United States using a validated bowel health questionnaire based on the Bristol stool form scale, reported a prevalence of 6.6% in the adult American population [2].

When dietitians are faced with patients with chronic diarrhoea, it is imperative that a diagnosis is pursued and that sinister diagnoses are excluded. This will involve referral to, and collaboration with, the patient’s General Practitioner or Gastroenterologist. Alarm features that suggest more worrying diagnoses include rectal bleeding, symptoms that wake the patient from sleep, unintentional weight loss, severe unremitting symptoms, a family history of inflammatory bowel disease or colorectal cancer and onset of new symptoms in a patient over the age of 50 years. However, patients without alarm symptoms may also need to undergo appropriate investigations.

Non-invasive investigations could include a full blood count, urea and electrolytes, coeliac serology, iron, vitamin B12 and folate, faecal culture and parasites. A measure of pancreatic exocrine function (e.g., faecal fat concentration or elastase) can be pursued if steatorrhoea is suspected. If indicated, colonoscopy with mucosal biopsy is the gold-standard test to diagnose serious ileocolonic pathology including colorectal cancer, microscopic colitis and inflammatory bowel disease. Other testing can be helpful in specific circumstances including hydrogen and methane breath testing for carbohydrate malabsorption and small intestinal bacterial overgrowth.

Once a diagnosis is made, or serious diagnoses are excluded, dietitians play a key role in the management of chronic diarrhoea. Often the cause may be functional and can lead to a diagnosis of functional diarrhoea (FD) or diarrhoea-predominant irritable bowel syndrome (IBS-D) (Table 1). The dietitian’s role varies depending on the underlying cause of the diarrhoea, with a wide range of dietary therapies available. However, dietitians also have an important role in educating patients about the perils and pitfalls of dietary therapy. Depending upon the cause, if left untreated, chronic diarrhoea may lead to malnutrition and micronutrient deficiencies [4]. Likewise, micronutrient deficiencies may occur when foods or food groups are removed to manage symptoms without considering diet adequacy [5]. Chronic diarrhoea may also impact on quality of life, with increased incidence of depression described in populations of both young [2] and older adults [6].

### 1.1. Diet Seeking Behaviour by Patients with Gastrointestinal Symptoms

Prior to seeing a health professional, patients often identify intolerance to specific foods or food groups, either by trial and error or through doing their own research. The internet has become a platform for seeking such advice [7]. A study comparing the advice provided in blogs by registered dietitians and non-dietitians (non-RD) such as certified holistic nutritionists, nutrition therapists, personal trainers and massage therapists found that non-RD most often provided specific nutrition advice on avoiding foods and promoting supplement use for health conditions including gut disorders [8]. The researchers also found that non-dietitian bloggers used fear-driven strategies and non-evidenced recommendations to support their advice. A survey of 1500 gastroenterologists at the American College of Gastroenterology found that almost 60% of patients seen had made dietary changes prior to their appointment with lactose-reduced and gluten-free diets being the most common [9]. A Swedish study of 197 IBS patients found that 84% reported at least one food as a trigger for their symptoms, with the number of foods identified as problematic increasing linearly in proportion to symptom severity [10]. Dairy and wheat products were commonly avoided [9,10]. 

This review addresses the diet-responsive causes of chronic diarrhoea, provides an evidence-based overview of diet-based therapies, explores the pearls and pitfalls of each therapy and outlines the role that dietitians have in ensuring the nutritional adequacy and safety of dietary changes.

### 1.2. Understanding the Role of Diet in the Management of Chronic Diarrhoea

**Figure 1:**
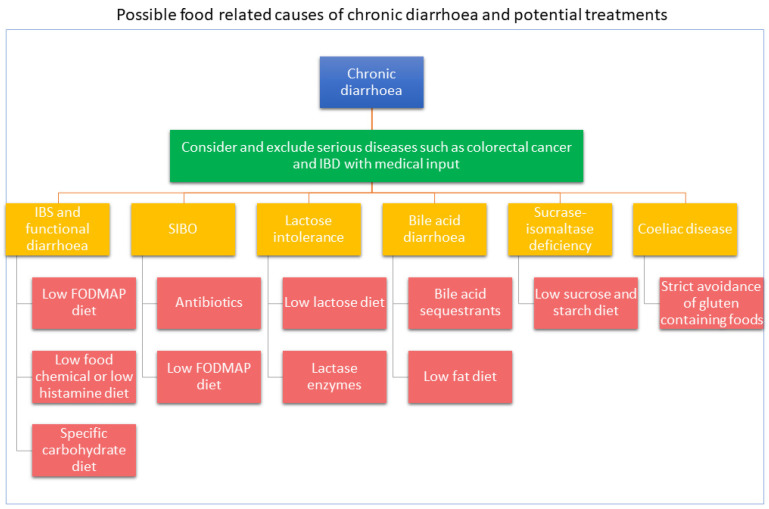
Evidence-based approaches for the management of chronic diarrhoea.

There is a wide range of common causes of diarrhoea that can be classified depending on their responsiveness to dietary therapy (Table 2). The mechanisms for causing diarrhoea are varied and provide the basis for evidence-based dietary interventions. Often dietary therapy is part of a multidisciplinary approach to treat diarrhoea and may include drugs, gut-directed hypnotherapy or stress management. Figure 1 describes evidence-based approaches for the management of chronic diarrhoea.

## 2. Dietary Management of Chronic Diarrhoea

### 2.1. Irritable Bowel Syndrome (IBS) and Functional Diarrhoea

Irritable bowel syndrome (IBS) affects 4–20% of the population [11]. Women are more likely than men to be diagnosed with IBS, and its prevalence, based on the Rome IV criteria (Table 1), is greater in the 30–45 year age group and for those living in Western countries [11]. IBS results in changes in bowel habits, bloating, pain and nausea. It also impacts energy levels [10] and quality of life [10,12] and is a common reason for visits to general practice [13], yet its aetiology remains unclear. IBS can persist for many years and develop at any age. A ten year follow-up study of over 8000 patients enrolled in a screening programme found that almost two-thirds of patients with IBS continued to have symptoms at follow up, while 28% of patients without symptoms at baseline subsequently developed IBS [14].

To diagnose IBS, abdominal pain must be present, and other gut-related causes eliminated such as coeliac disease, inflammatory bowel disease, colorectal cancer or diverticulitis. The Rome criteria classifies IBS as diarrhoea predominant (IBS–D), constipation predominant (IBS–C), mixed bowel habits (IBS-M) or unclassified (IBS-U) [15]. FD is diagnosed in those with diarrhoea but not abdominal pain.

#### 2.1.1. Dietary Therapies for IBS

First-line dietary advice such as eating regularly, limiting intake of high-fibre food, reducing the intake of alcohol, caffeine, fizzy drinks and managing stress is sufficient to resolve IBS symptoms for up to 50% of patients [16,17]. If symptoms persist, a trial of an elimination diet to identify specific foods that trigger IBS symptoms may be warranted. There are a number of elimination diets described in the literature. The following sections outline three elimination diets, the evidence to support their use and the pearls and pitfalls of each dietary treatment (Table 3). Non-coeliac gluten sensitivity, which is often reported by patients, remains difficult to diagnose and define [18]. Patients may be reacting to other components within food such as fermentable carbohydrates [19,20]. Because this diet therapy is currently unproven it is not included in this review.

#### 2.1.2. The Low FODMAP Diet

The low fermentable oligosaccharides, disaccharides, monosaccharides and polyols (FODMAP) diet comprises three phases: elimination, reintroduction and then liberalisation or personalisation of the diet. The elimination phase of the low FODMAP diet effectively relieves symptoms in 50–85% of patients with IBS-D [21] and is the primary dietary treatment for IBS not responsive to first-line dietary advice [16,22]. The low FODMAP diet reduces fermentable carbohydrates that elicit an osmotic effect by drawing fluid into the small intestine and reducing colonic fermentation and gas production by the indigenous colonic bacteria [23]. For those with IBS, visceral afferent hypersensitivity results in bloating and pain.

Numerous observational, randomised control trials [24,25,26,27,28,29,30] and meta-analyses [31,32,33,34] support the efficacy of a low FODMAP diet to treat adults with IBS. Prospective and observation studies across all age groups have also been conducted [35,36]. The low FODMAP diet is a short-term restrictive diet; the reintroduction and long-term dietary management are important to ensure diet diversity [37]. There are published protocols, based on expert opinion, for the reintroduction phase of the low FODMAP diet [38], which should be conducted four-six weeks after phase 1. To help patients navigate the low FODMAP diet, there are designated websites, smart phone applications, social media pages, books, magazines and lists of foods high and low in each FODMAP group that are regularly updated. However, dietary instruction should only be given by clinicians with “expertise in dietary management” [17], ideally a dietitian, as dietary compliance is more likely to occur, especially in phase 2 of the diet [39]. Total avoidance of food groups is not required, tolerance levels can vary greatly, with some patients able to manage fairly large amounts of restricted foods and others only limited quantities. The low FODMAP diet allows for a less restrictive approach than a strict avoidance of all gluten or dairy containing products, which is often trialled by those with chronic diarrhoea [9]. Foods that contain wheat as a minor ingredient can be eaten during the elimination phase. Likewise, only high-lactose dairy is avoided; products such as butter, cheese, and small amounts of milk are generally well tolerated, allowing more food choices (Table 3).

A major pitfall of the diet is the length of time to establish “safe” foods that are unlikely to trigger symptoms. Restricting food for unnecessarily long periods increases the risk of nutritional deficiencies or changes in the gut microbiome occurring and the potential for unhealthy relationships with food developing [24,26,27]. A RCT by Staudacher et al. measured absolute Bifidobacterium species abundance for those undertaking phase one of the low FODMAP diet blinded to taking a probiotic or placebo [27]. The low FODMAP diet reduced Bifidobacterium species, but for those taking probiotics containing Bifidobacterium strains, an increase in Bifidobacterium species abundance occurred. Results have varied from subsequent studies with some groups finding alterations in the bacteria studied and others no change [40]. Regardless, the low FODMAP diet reduces the choice of prebiotic foods from the diet. This is a potential pitfall as these important bacterial substrates lead to the production of beneficial short-chain fatty acids (SCFA). Maintaining a diet higher in resistant starches and non-starch polysaccharides that are less likely to lead to excessive gas production, during phase one of the low FODMAP diet, could buffer the effects on the gut microbiota [41].

IBS may be associated with disordered eating patterns such as Avoidant or Restrictive Food Intake Disorder (ARFID) [42]. There is also a high prevalence of functional diarrhoea in those who are diagnosed with eating disorders [43]. A more conservative approach is now advocated for high-risk groups [44], as fewer foods are restricted [44,45]. A modified or “FODMAP-gentle” approach reduces foods that are most likely to be problematic such as wheat, onion, leek, apples, pear, milk and legumes [44], thus providing more diet variety and less focus on restriction. Further restriction, however, may be needed if symptoms do not improve, potentially prolonging phase 1. A thorough dietary assessment may indicate which approach will be of most benefit.

It should also be acknowledged that the FODMAP content of foods has been mostly measured in Australian foods, and limits set with the best estimates from a small group of individuals [46]. There are likely to be international differences in FODMAP content and FODMAP tolerance may differ between people; therefore, an individualised approach will always be best. However, of all the dietary interventions for the management of IBS-D, the low FODMAP diet is the most studied option and is its use is recommended over and above other diet therapies [16,17].

#### 2.1.3. The Specific-Carbohydrate Diet

The specific-carbohydrate diet (SCD) allows mainly monosaccharide sugars (fructose, glucose, galactose) but restricts disaccharides (lactose, sucrose, maltose) and most polysaccharides (starch). Accordingly, most ripe fruits and vegetables are allowed, but all grains are excluded. Similarly, disaccharide-rich foods such as lactose-containing dairy products, sucrose-sweetened foods and beverages as well as food additives and preservatives are restricted. The use of dairy products that are low in lactose such as long fermented yoghurt is permitted. Patients are advised to re-introduce restricted foods once symptoms resolve but the length of time required is poorly defined.

It has been proposed that much like the low FODMAP diet, the mechanism behind the diet is to alter the gut bacteria to restore balance [47] and reduce the by-products of food metabolism within the bowel that induce unwanted symptoms [48]. Unlike the low FODMAP diet, however, there is scant scientific evidence to support the SCD rationale.

The diet was initially developed in the 1950s to treat coeliac disease. It was popularised by publication of the book Breaking the Vicious Cycle [48] in the 1990s and has since been used to treat various gastrointestinal conditions. There are online SCD food lists and guidelines; however, the dietary information is often contradictory. The evidence to date on the effectiveness of the SCD for adults is limited and mostly conducted in those with inflammatory bowel disease (IBD) [48,49,50]. Research in IBS is scarce. To date, only one RCT of 60 patients with IBS has been published. Patients were randomised to either three months of phase 1 of a low FODMAP diet or the SCD. Symptom improvement was measured using the IBS-SSS (Irritable Bowel Syndrome Severity Score). There was a non-significant reduction in bloating and distention in the SCD group, and the diet was not as effective as the low FODMAP diet [51]. Previous SCD research in IBD patient cohorts suggests that symptom improvement may take 12 months [48,49] in which case the study duration of three months may have been insufficient to induce symptoms improvement in the IBS study participants.

**Figure 2:**
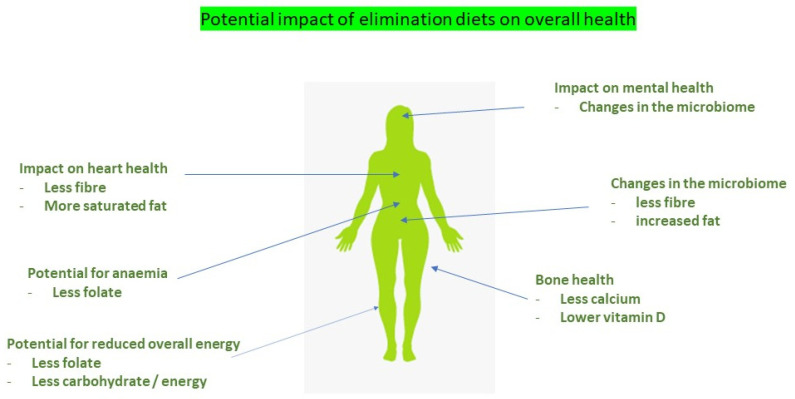
The potential impact of elimination diets on overall health.

The SCD restricts many foods as well as the bread and cereals. As a result, deficient intake of specific macronutrients (e.g., fibre, carbohydrate) and micronutrients (B1, vitamin D, calcium) is possible [52]. If not carefully managed this diet could negatively impact on diet adequacy, quality of life or mental health. Table 4 provides management strategies for potential pitfalls. Due to the number of food restrictions and length of time for symptom reduction the development of disordered eating is also possible. Restricting dietary fibre is especially concerning [53] (Figure 2). At a population level, low fibre intake has been associated with the increased risk of colorectal cancer [54], cardiovascular disease [55], obesity [56] and type 2 diabetes [57]. At a microbiota level, changes in the diversity and abundance of bacteria have been observed with changes in high-fat diets [58] or high-protein foods [59], both likely to occur when carbohydrates are restricted. SCD diet advice should be provided by a dietitian experienced in its delivery and focus on the inclusion of as many high-fibre foods as possible, as well as addressing the potential for dietary inadequacy.

#### 2.1.4. The Low-Food Chemical Diet/Low-Histamine Diet

Case reports suggest that chronic diarrhoea improves with the reduction in bioactive food chemicals such as amines, glutamates, salicylates and histamine. There are two schools of thought on the role of bioactive food chemicals to treat chronic diarrhoea. The low-histamine approach is advocated as a treatment for mast cell activation syndrome (MCAS) [60], and the low-food chemical approach, developed at the Royal Prince Alfred Hospital allergy unit in Sydney, Australia and commonly known at the Royal Prince Alfred Hospital (RPAH) diet, is used to treat chronic diarrhoea in the presence of non-gastrointestinal-related symptoms including urticaria, asthma or rhinosinusitis [61]. The prevalence of food chemical or histamine intolerance is unclear. It is suggested that the prevalence is underestimated and may affect 20% of those with food intolerance [62]. It has also been proposed that histamine intolerance is associated with the genetic condition Ehlers-Danlos syndrome (EDS) [63].

Unlike IgA food allergies in which symptoms typically occur shortly after exposure [64], food chemical intolerance symptoms are described as occurring within minutes or may not occur until the following day [65]. The delayed onset of symptoms is thought to be related to a threshold effect. The accumulation of a substance in the body over the day or several days [66] stimulates a gastrointestinal neuroendocrine system response. This delayed response makes it difficult in both a research and clinical setting to determine the cause and effect relationship between symptoms and dietary intake or other factors.

Histamine intolerance is driven by reduced histamine degradation resulting in histamine accumulation [67]. A reduction in the enzyme diamine oxidase (DAO), needed to degrade histamine, along with dietary sources of histamine-containing foods results in an overload of histamine, resulting in symptoms, including diarrhoea [60].

The RPAH diet was first described in 1978 to treat chronic urticaria [68] and subsequently to manage gastrointestinal symptoms due to food intolerances [65]. There are no published observational or interventional studies of the RPAH to treat IBS, although there is commentary in the literature [61,69,70]. The diet adequacy of patients following the RPAH diet has been reported and can be viewed on the RPAH website [71]. However, these reports all come from one clinic and may not be representative. Anecdotally the RPAH diet is used in clinical practice and is supported by the RPAH published handbook [72]. The RPAH diet may be effective, but given the lack of published research, it has been recommended that the elimination and subsequent systematic food re-challenge only be conducted by clinicians trained in the delivery of the diet and is best reserved as a second-line dietary therapy [73]. In some cases, symptom improvement may be due to lifestyle changes and less processed and more whole foods intake rather than the suspected food chemical [70].

Salicylates, one of the food chemicals in the RPAH diet, are thought to be the most likely natural food chemical to cause symptoms [62]. It is suggested that salicylates increase smooth muscle contraction within the gastrointestinal (GI) tract [74]. However, research into the effects of dietary salicylates is limited, and is extrapolated from non-steroidal anti-inflammatory drug (NSAID) intolerance. Cuomo et al. have suggested that salicylate intolerance could be a diagnosis of elimination after all other possible causes are excluded [75].

Despite a lack of high-quality evidence, patients often report that foods rich in bioactive chemicals, such as salami, cheese, wine and beer, are dietary triggers of their symptoms [10]. However, the symptom reduction reproducibility from following a low-histamine diet has been questioned. A clinical trial reported that participants undertaking a low-histamine diet reacted “unexpectantly and randomly” when challenged with a placebo low-histamine tea versus tea containing liquid histamine [76]. However, the placebo tea contained peppermint, a high salicylate food that could have also triggered symptoms.

Tuck et al. provide a pathway for diagnosing histamine intolerance in their review, based on a three-phase diet and symptom response, similar to the three phases of the low FODMAP diet: 1st phase, strict low-histamine diet; 2nd phase, the specific reintroduction of histamine-rich foods; 3rd phase, long-term diet modification adapted to the individual [67]. This approach would work equally well with the RPAH diet. The diets should be adapted to best suit the individual. When followed in their entirety, they can be highly restrictive. Some patients may only need to reduce foods containing very high levels of natural food chemicals to obtain a benefit.

A pitfall of any food elimination diet is the accuracy of which foods are recommended for inclusion and exclusion (Table 3). In the case of salicylates, a 2017 study found that for some foods, especially spices, the content of salicylate when tested using advanced analytical methods was different compared to foods previously tested between 1985 and 2011 [77]. This new research may make many of the existing food lists obsolete, but also highlighted how the amount of salicylates can vary depending if the food is peeled or unpeeled, as with the case of apples, fresh or dried, e.g., basil leaves, or between different varieties of a fruit such as grapes and plums.

As with other dietary therapies, if the restriction period is not carefully managed there is risk of overly restricting dietary fibre. The risk of this is likely greater for the RPAH diet due to limited fruit with only pears and vegetables allowed if the strictest protocol is followed. Similarly, due to the restrictive nature of elimination diets, there is a risk of vitamin deficiency, especially vitamin A and folate. The restriction reintroduction phases, therefore, should be supervised under expert guidance.

### 2.2. Small Intestinal Bacteria Overgrowth (SIBO)

Small intestinal bacterial overgrowth (SIBO) is caused when there is an overgrowth of the naturally low number of bacteria present in the small intestine [80]. The symptoms of SIBO include diarrhoea, bloating, abdominal pain and flatulence [80], and may result in altered nutrient levels such as elevated folate, vitamin K and reduced vitamin B12 through the bacterial consumption of vitamins and cleavage of the B12-intrinsic factor bond [81]. Several aetiologies for SIBO are proposed. These include changes in how the small bowel cleanses itself through migratory motor complexes, anatomic alterations including small bowel diverticulosis or fistulas [82] and a change in the pH of the small intestine allowing bacteria to colonise [80]. There has been much debate as to whether SIBO occurs due to underlying GI conditions, or is a condition in itself, due to factors such as altered small bowel motility [83].

SIBO may be an under-recognised factor in patients with chronic diarrhoea. In a study of 87 patients experiencing chronic diarrhoea, SIBO, diagnosed on either jejunal aspirates or hydrogen breath tests, was more likely to be present (48%) than other causes such as IBS (13%), microscopic colitis (6%) or infections (6%) [84]. It should be noted, however, that the diagnosis of SIBO through hydrogen breath testing is limited. Diagnosis of SIBO, in those with IBS can vary quite considerably due to the differences in cut-off values and inconsistent methodologies [85]. Clinical guidelines suggest weak evidence for the use of hydrogen breath tests and that cut off values should be standardized [86].

As yet, there is no gold-standard pharmaceutical or dietary treatments for SIBO. The most common pharmaceutical treatment a non-systemically absorbed antibiotic, Rifaximin, which locally targets overgrowth of bacteria in the small bowel [87]. A meta-analysis by Gatta et al. found that Rifaximin had a 70% success rate for treating SIBO in almost 1200 patients [87]. Effectiveness may be greater when insoluble fibre is added to the treatment, as seen in a study using partially hydrolysed guar gum (PHGG) [88]. In this study, eradication rates for SIBO were significantly greater in the group treated with Rifaximin and PHGG (34/39 patients, 87%) compared to those treated with Rifaximin alone (23/37 patients, 62%) *p* = 0.0017. Unfortunately, reoccurrence of diarrhoea symptoms is common, in up to nearly half of patients at nine months [89]. Although there is no consensus, the American College of Gastroenterology guidelines for SIBO recommend that if SIBO reoccurs, rotation of different types of antibiotics is required to manage symptoms and prevent antibiotic resistance [86].

Some practitioners also use herbal treatments for the treatment of SIBO. An intervention comparing the use of herbal antibiotic treatment to the antibiotic Rifaximin for those with SIBO, as measured by improvements in hydrogen breath testing, found that the herbal antibiotic was more effective than Rifaximin (17/37 or 45.9% vs. 23/67 or 34.3%) although the differences were not statistically significant (*p* = 0.24) [90]. No data on symptom improvement were reported. However, only 400 mg of Rifaximin was given three times a day (TDS), whereas the latest recommendations are for 550 mg TDS [86] and this may account for the low response rate to Rifaximin. Dietary restrictions such as following the low FODMAP diet, or the specific-carbohydrate diet have been anecdotally reported. Replacing all food with a two-week trial of elemental drinks has also been studied [91]. However, evidence to support these dietary treatments is limited. In the absence of a gold standard for diagnosis and treatment of SIBO, the British Society of Gastroenterology guidelines for the investigation of chronic diarrhoea in adults recommend proceeding to an empirical antibiotic trial when SIBO is suspected [1]. Antibiotic treatment will depend on the type of bacterial overgrowth occurring, as the recommended treatment for bacteria producing methane (methanogen overgrowth) differs from overgrowth from bacteria that predominately produce hydrogen [81].

### 2.3. Lactose Intolerance

Lactose malabsorption is due to the absence or inadequate production of the digestive enzyme lactase, which is required to digest the disaccharide lactose in the small intestine, causing lactose intolerance to occur. Lactose intolerance may result in abdominal pain, diarrhoea and flatulence [92]. Lactose malabsorption occurs in those that do not carry the inherited genetic trait of lactase persistence and is most prevalent in Asian and African countries. In contrast, north western European populations, such as Sweden, Holland and the United Kingdom, lactose malabsorption affects less than 20% of the population [93]. The prevalence of lactose malabsorption is the same for healthy and IBS populations, but those with IBS are more likely to be intolerant due to visceral afferent hypersensitivity and altered gut transit [94]. Gene or hydrogen breath testing is used to diagnose lactose malabsorption [95,96], with avoidance of lactose and a subsequent reintroduction to confirm a diagnosis [97]. If lactose intolerance is present, total avoidance of dairy is not required, the degree of enzyme activity varies, up to 12 g of lactose, equivalent to a 250 mL glass of milk, can be tolerated by some [98]. It is also likely that lactose-containing foods may be better tolerated when eaten as part of a meal rather than in isolation [96]. A recent cross-over RCT of 29 IBS patients found that both a low FODMAP diet and a low-lactose diet significantly improved symptoms [99]. A thorough dietary review would guide such a decision.

Patients often restrict dairy due to the self-belief that they are dairy or lactose intolerant [100,101]. In a study of over 900 Chinese patients only 58% of those with self-reported lactose intolerance were diagnosed with lactose malabsorption [102]. This leads to the unnecessary restriction of dairy products which can lead to calcium and vitamin D deficiency, both being important to maintain bone health. [103] It is recommended that all patients, self-reported and diagnosed, reintroduce lactose products to determine their tolerance, replace dairy foods with calcium fortified alternatives or consider calcium supplementation or fortified foods [95]. Alternatively, lactase enzymes could be ingested to enjoy the benefits of milk without experiencing symptoms of lactose intolerance.

### 2.4. Bile acid Diarrhoea (BAD)

Bile acid diarrhoea (BAD), also known as bile acid malabsorption, occurs when bile acids that are secreted through bile are not reabsorbed in the terminal ileum via the enterohepatic circulation and continue to the colon [104]. This results in increased gut motility and colonic fluid, leading to watery diarrhoea that is pale in colour and often difficult to flush [105]; less common symptoms are bloating, pain or increased flatulence [106]. Diagnosis may be made using either a radiolabelled synthetic Selenium-homocholic acid conjugated with taurine (SeHCAT), a C4 assay or an empirical trial of bile acid sequestrants. If there is an underlying and treatable cause of BAD, this will also need to be addressed.

BAD is thought to be under-diagnosed and may affect up to 41% of patients presenting with IBS-D [107,108]. Bile acid diarrhoea is suggested to be a possible cause of chronic diarrhoea in those who do not respond to dietary and lifestyle changes [1]. It has been proposed that screening for BAD should occur prior to a diagnosis of IBS being made, as currently takes place for coeliac disease, especially for patients who experience chronic diarrhoea following bowel surgery or cholecystectomy [106]. However, testing is not always readily available and a therapeutic trial of a bile acid sequestrant may be more feasible.

Bile acid sequestrants are the first-line treatment for BAD, but diarrhoea may still occur after consuming high-fat meals. A UK prospective study of 40 individuals diagnosed with BAD found that a reduction in dietary fat to 20% of total energy (approximately 40 g or less per day) was an effective treatment, both for those taking and not taking bile acid sequestrants [109]. This approach may be an option for those not wanting to take daily medications, that are not always tolerated due to the grainy texture and taste [110]. However, dietary fat is also required for the absorption and metabolism of fat-soluble vitamins [111].

A low-fat diet may lead to inadequate intake of fat-soluble vitamins (vitamins A, D, E and K) resulting in nutritional deficiencies, fat is also needed to move the vitamins out of the small intestine to be circulated throughout the body. Fat-soluble vitamins are found naturally in a number of foods ranging from fruits, vegetables, plant and animal fats. They are cleaved from food in the small bowel by bile and digestive enzymes and then transported to the liver, muscles or adipose tissues for storage [111]. Bioavailability of fat-soluble vitamins is impacted by genetic factors, age, sex and disease state; some individuals may need more than the recommended daily intake and some may need less [112]. This could be a potential pitfall of BAD or dietary management of BAD through a low-fat diet. There is no consensus on the need to monitor serum/plasm fat-soluble vitamins [113] although screening for deficiency, if suspected, is prudent in the management of BAD. For those wanting to manage symptoms with diet, dietetic involvement would be required to educate the patient on sources of fat in food, both obvious and hidden, and to ensure their diet’s nutritional adequacy, particularly for fat-soluble vitamins.

### 2.5. Sucrase-Isomaltase Deficiency (SID)

Foods containing starch, such as grains, potatoes and other starchy vegetables and some fruit, need to be digested into monosaccharides to be absorbed. The digestive process starts in the mouth with salivary α-amylase and continues in the small intestine. Pancreatic amylase digests starch into disaccharides maltose and dextrin and then in the brush border sucrase-isomaltase completes the digestion process [114]. The gold standard to diagnose SID is duodenal biopsy [115]. Genetic testing can also be used; however, not all the sucrase-isomaltase gene mutations have been identified; therefore, a negative gene test cannot be used to rule out SID [116]. Deficiencies in these enzymes may be due to genetic polymorphisms, mostly diagnosed in childhood, but possibly not identified until adulthood. Secondary SID can arise from damage to the duodenal villi from coeliac disease, infections or SIBO [116].

Disaccharide enzyme deficiencies result in the undigested disaccharides moving through to the large bowel drawing fluid as they go resulting in diarrhoea [117]. The malabsorbed disaccharides are fermented by large bowel microbiota the by-product of which is excess gas production, leading to bloating and pain [21] Thus, the symptoms of disaccharide enzyme deficiency can often be misdiagnosed as IBS [116]. In an existing cohort of 46 participants with IBS-D, symptoms were re-examined. Those who carried sucrose-isomaltase genetic variants were less likely to experience symptom relief with the low FODMAP diet than non-carriers of the variants (43.5% vs. 60.9%, *p* = 0.031) [118]. The baseline clinical characteristics were not significantly different between the non-carrier and carrier groups. A study of 2207 IBS patients across Europe and the United States found that 4.2% of those with IBS-D were carriers of rare sucrase-isomaltase pathogenic variants [119], illustrating that some IBS patients may have poorly functioning digestive enzymes, resulting in symptoms of IBS caused by SID. A case control study of those with and without IBS found that those with IBS were more likely to have a genetic mutation for sucrase deficiency [120].

Pharmaceutical options are available, but the restriction of high starch carbohydrates is the primary dietary strategy to manage SID [121,122] despite the lack of clinical studies. An RCT of 105 patients with IBS randomised patients to follow a starch- and sucrose-reduced diet or continue with their usual diet. Those in the intervention group experienced significant and rapid improvements with symptoms and quality of life. There are few published studies on the foods to avoid and those safe to eat other than websites referenced in the RCT [123] and education guidelines [122]. It is possible that those with SID also experience lactose deficiency, as reported in a study of 31 patients with presumed IBS: 35% were diagnosed with SID and all those with SID also were diagnosed with lactase deficiency based on duodenal biopsies [116]. Lactose malabsorption could make the diet even more limiting. Chewing food well and combining carbohydrates with foods containing protein and fat to slow digestion are also considered important to allow time for sucrose to be hydrolysed [123].

Given that secondary SID has been suggested to be more common than primary, the possibility that those with chronic diarrhoea have SID is worth considering, but clinicians need to be aware that published, peer-reviewed data are lacking. Screening for genetic morphisms, rather than symptoms alone, may enable patients to access therapies that best suit the underlying cause of chronic diarrhoea. [118].

### 2.6. Coeliac Disease

Coeliac disease is an immune condition that results in chronic inflammation of the small bowel after digestion of gluten [124]. The disease is hereditary, with 40% of the world’s population carrying the HL-DQ2 or HL-DQ8 gene. However, prevalence is approximately 1%. Therefore, environmental factors also play an important role [124].

The only current treatment for coeliac disease is the strict avoidance of all gluten. The consumption of even small amounts of gluten results in histological changes to the small bowel villi reducing the secretion of enzymes and lumen surface area to absorb micronutrients such as folate, iron and vitamins B12 and D, and leading to GI symptoms [125]. Additionally, if a gluten-free diet is not nutritionally balanced there is a risk of inadequate intake of fibre, folate and B vitamins [126], nutrients commonly found in grains and cereals. Consequently, screening for micronutrient deficiencies should be conducted at diagnosis and periodically.

A pitfall of lifelong gluten-free dietary treatment is that strictly following the diet means reading food labels and being aware of gluten-containing ingredients in food, medicines, supplements, and eating out. The identification and cost of gluten-free foods may reduce dietary compliance [127,128]. In a Canadian study, patients with coeliac disease were sent questionnaires about following a gluten-free diet, and only 49% were able to correctly identify gluten-free foods listed [129]. Dietary education is a predictor of adherence to following a strict gluten-free diet [127], and dietary adherence has been reported to be associated with better quality of life [130]. Clinical guidelines recommend all newly diagnosed patients be seen by a dietitian [131] and a systematic review of 38 studies, found that dietary information provided by dietitians was valued by patients more than that of other health professionals [132]. However, in a study of almost 6000 individuals with coeliac disease, only 50% rated the information received from the dietitian as being very good or excellent, compared to information from the internet (53%), cookbooks (62%) and word of mouth (66%) [129]. It is unknown if patients in this study were seen by a dietitian experienced with managing coeliac disease. Dietitians need to remain up to date and provide dietary information that is appropriate and tailored to the patient’s needs.

## 3. Dietary Role of the Dietitian

Patients diagnosed with IBS, functional diarrhoea or SIBO are most likely to be exposed to conflicting and confusing information. For these groups, there are a plethora of dietary recommendations, many of which lack sound evidence, and often patients are advised by their doctor to search on-line for information or only basic information is provided [133]. Commonly, patients have already tried dietary changes, and at times these recommendations have been followed unnecessarily for long periods, resulting in overly restrictive diets. Both evidence-based and non-evidenced-based information could have negative impacts on overall health (Figure 2).

Dietetic intervention at the time of diagnosis will help to mitigate the pitfalls of ill-informed dietary advice. A review of IBS patients recommended to follow the low FODMAP diet by various health professionals or through word of mouth found that when a dietitian provided the information, patients achieved better outcomes during phase 2 (the re-challenge phase) and phase 3 (the long-term maintenance phase) than when the diet was recommended through other avenues [39].

Dietitians are the experts on the nutritional management of conditions where food plays a role, including chronic diarrhoea. As seen with the low FODMAP diet, dietitians also have an important role in research [23,25,26,29,35,36,73,134] and the development of evidence-based practice [16]. Thus, the role of the dietitian is multifactorial and extends beyond one-on-one nutrition counselling. For direct patient care, however, dietitians can provide nutritional counselling that guides patients through their continuum of care (Table 5).

## 4. Conclusions

The pearls of dietary management for chronic diarrhoea are improving digestive symptoms, energy levels, reduced reliance or need for pharmaceutical medications and increased quality of life. However, without proper instruction or management, the restrictive nature of diets could reduce diet quality such as less fibre, and fewer calories or micronutrients. The pitfalls of such changes, especially reducing carbohydrates and thus fibre, is the impact upon the gut microbiota and subsequent overall health. It is essential that clinicians bear this in mind and provide recommendations that minimise any negative impact.

## Figures and Tables

**Table 1:** Rome IV criteria for irritable bowel syndrome and functional diarrhoea.

Rome IV Criteria for Irritable Bowel Syndrome–D, M	Rome IV Criteria for Functional Diarrhoea
Abdominal pain on average at least 1 day/week in the last 3 months that is associated with at least 2 of the following	Not usually associated with pain
Related to defecation Change in stool frequency Change in stool form	Loose or watery stools at least 25% of the time
Duration of more than three months	Duration of more than three months

**Table 2:** Common causes of chronic diarrhoea—pharmaceutical and dietary responsive.

Common Causes of Chronic Diarrhoea	Mechanism	Dietary Management
**Predominantly pharmaceutical responsive**		
Pancreatic insufficiency	Insufficient secretion of pancreatic digestive enzymes into the small intestine	Teaching patients sources of fat so they are able to titrate digestive enzymes effectively
Microscopic colitis	Inflammation occurring at a microscopic level in the lining of the large intestine	N/A
**Combination of pharmaceutical and dietary responsive**		
Short-bowel syndrome	Reduced mucosal surface due to removal or damage of part of the small intestine	Dietary manipulation to enhance absorption such as small frequent meals, higher protein and less refined sugar
Inflammatory bowel diseases	Chronic intestinal inflammation occurring throughout the gastrointestinal tract	Dietary and nutrition therapies to manage inflammation and promote maintenance of remission
Small intestinal bacterial overgrowth	Overgrowth of colonic bacteria in the small intestine	Restriction of fermentable carbohydrates (the low FODMAP diet) or an elemental diet may reduce overgrowth if antibiotics have not been responsive
Bile acid diarrhoea	Excess bile acids entering the large intestine	A low-fat diet may reduce the production of bile acids
**Predominantly dietary responsive**		
Irritable bowel syndrome	Mechanisms are not clearly understood but could be due to increased gut transit, visceral hypersensitivity or altered gut microbiome	Dietary strategies could include: reducing portion sizes, regular eating, reducing fermentable carbohydrates or reducing natural food chemicals
Lactose intolerance	Reduced lactase enzyme activity in the small intestine	Limiting lactose-containing milk and milk products
Sucrase-isomaltase intolerance	Reduced enzyme activity of sucrase and or isomaltase in the small intestine	Reducing dietary intake of foods containing sucrose, isomaltose and maltose
Coeliac disease	Genetic condition resulting in damage to the lining of the small intestine when gluten is consumed	A strict lifelong gluten-free diet resolves symptoms and results in healing the lining of the small intestine

**Table 3:** The pearls and pitfalls of dietary therapies for the management of chronic diarrhoea.

Disease	Dietary Therapy	Pearls	Pitfalls
Irritable bowel syndrome (IBS)	Low FODMAP diet	The most studied dietary intervention across all age groups.	The long length of time to establish likely trigger foods.
		There are multiple resources; designated websites, apps, recipes, Facebook pages, books, magazines.	Obsolete and outdated information is likely; resources need regular review by qualified health professionals.
		Comprehensive dietitian training is available.	FODMAP content differs by country. Individual tolerance may differ.
		Commercial product FODMAP testing is available increases consumer choice.	Phase 1 may restrict prebiotic food intake.
		A modified version can be used with those at high risk.	Restrictive diets may contribute to disordered eating patterns.
		Small amounts of wheat are allowed so a gluten-free diet is not required.	Phase 1 may reduce abundance of multiple bacterial species.
		High-lactose dairy is avoided. A dairy free diet is not required.	
	Specific-carbohydrate diet	*Breaking the Vicious Cycle* book provides detailed instruction.	Limited evidence of mechanisms, food composition and efficacy.
		Online support is available.	Long length of time to achieve improvements.
			No evidence of impact on diet adequacy, quality of life and mental health.
			Limited and conflicting guidance on use of the diet and reintroducing foods.
			Restrictive diets may contribute to disordered eating patterns.
			Likely restricts prebiotic food intake and nutrient intake.
	The low-food chemical/low-histamine diet	The Royal Prince Alfred Hospital provides detailed instruction for the low-food chemical diet.	Limited evidence of efficacy.
		There are multiple resources; designated websites, apps, recipes, Facebook pages, books.	Limited and conflicting food chemical content data.
		Relatively short elimination period.	Triggers may be non-diet related.
		A modified version can be used with those at high risk.	Likely restricts prebiotic and nutrient intake.
		May address a wider range of intolerances.	Restrictive diets may contribute to disordered eating patterns.
Small intestinal bacteria overgrowth (SIBO)	Low FODMAP diet	Excellent support information available.	Online information is prevalence, but given the lack of evidence in this field, it is likely to lack any validity.
Dietary changes may not be needed if antibiotics are effective			Reoccurrence of SIBO is common, risking nutritional deficiencies if repeated dietary restriction is conducted.
	Elemental diet	Nutritional complete	Provides no fibre and restricts prebiotics.
		Patients may not require any dietary restrictions.	May not be palatable and therefore poorly tolerated.
Lactose intolerance	Low-lactose diet	Credible methods for diagnosing are available.	Lactose-free products or lactase enzymes may not be easily available or affordable for all.
		Suitable alternatives are available providing nutrition in similar amounts.	Risk of low intake of calcium and vitamin D.
		High-lactose dairy is avoided. A dairy free diet is not required.	
Bile acid diarrhoea	Low-fFat diet	May be better tolerated than bile acid sequestrants.	Risk of inadequate intake of fat-soluble vitamins and reduction in overall energy intake leading to unintended weight loss.
Dietary changes may not be needed if bile acid sequestrants are effective		A variety of low-fat products are readily available at same cost to the full fat varieties.	
Sucrase-isomaltase deficiency (SID)	Low-sucrose/starch diet	There are multiple resources; designated websites, apps, recipes, Facebook pages, books.	Limited research on the long-term management of dietary changes.
		Oral enzymes are available to allowing for a broader range of foods to be eaten.	Sucrose enzymes are not available in all countries.
		With good planning the diet can still provide adequate fibre.	May restrict prebiotic food intake.
			Limited research on the long-term management of dietary changes.
Coeliac disease	Gluten-free diet	Gold standards for diagnosis.	Lifelong avoidance of all gluten-containing food is required.
		Gluten-free food alternatives are readily available.	Cross contamination can occur.
		There are multiple resources; designated websites, apps, recipes, Facebook pages, books.	Gluten-free alternatives can be more expensive, reducing diet compliance for some.

**Table 4:** Pitfalls of the dietary management of chronic diarrhoea and management strategies.

Potential Pitfall	Management Strategy
Unnecessary use of restrictive diet	Rule out other potential causes such as IBD, coeliac disease, diverticular disease, colorectal cancer [17]
	Consider general lifestyle and dietary advice first such as the NICE guidelines [17]
	Diagnostic testing to rule out SIBO and lactose malabsorption if available
Nutritional deficiencies	Review oral intake prior to commencing diet to determine if any already existing nutrient deficiencies
	Discuss suitable food alternatives
	Consider nutritional supplements for likely nutrient deficits
Diet restrictiveness	Consider lifestyle and general dietary advice first, e.g., NICE guidelines [17]
	Consider a modified version of the diet [44,45]
	Discuss food swaps where examples of food alternatives are given for each suggested eliminated food
	Develop a personalised plan during dietary eliminations [78]
	Provide shopping lists of suitable alternatives
	Provide recipe ideas and discuss meal planning
	Reintroduce restricted foods in a timely manner if improvements with symptoms or advise return to usual diet if not improvement was experienced
	Develop a personalised plan to include previously restricted foods that have been tolerated during the reintroduction phase
	Encourage frequent reintroduction of identified trigger foods, if appropriate, to test if threshold tolerance has increased
Changes in the microbiome	Promote diet diversity to prevent reducing fermentable fibre [79], encourage allowed foods that may not have been eaten before starting the diet
	Encourage vegetables or fruit at all meal times, pectin-containing fruit and vegetables may be better tolerated prebiotics [79]
	Encourage a fibre supplement if fibre intake is likely to be low [22]

**Table 5:** The role of the dietitian in the management of diet-responsive chronic diarrhoea.

Pre-Dietary Intervention
Remain up to date in the dietary management of chronic diarrhoea
A thorough assessment of current dietary adequacy
Review of medical history, gastroenterologist reports, blood tests, medications
In consultation with the patient, determine which dietary intervention is most appropriate
Careful instruction on how to follow the diet considering the pitfalls of the recommended dietary therapy (Table 4)
Determining if nutritional deficiencies are likely
**Dietary Support**
Explanation of the mechanisms of the diet and why dietary changes are required
Provision of recipes and menu plans if needed
Provision of diet alternatives than can replace nutrients from restricted foods
**Post-Dietary Intervention**
Review of effectiveness of dietary change
Review of diet adequacy
Instruction on how to reintroduce restricted foods if appropriate
Instruction on how to modify the diet for long term use, if needed
Instruction on ensuring diet diversity to minimise any likely nutrient deficiencies

## Data Availability

Not applicable.
